# Sports Activity and Patient-Related Outcomes after Cementless Total Hip Arthroplasty in Patients Younger than 40 Years

**DOI:** 10.3390/jcm10204644

**Published:** 2021-10-10

**Authors:** Luis Navas, Jasmin Faller, Sebastian Schmidt, Marcus Streit, Matthias Hauschild, Alexander Zimmerer

**Affiliations:** 1 ARCUS Sportklinik, Rastatterstr. 17-19, 75179 Pforzheim, Germany; navascontreras@sportklinik.de (L.N.); faller@sportklinik.de (J.F.); sebastian.schmidt@sportklinik.de (S.S.); streit@sportklinik.de (M.S.); hauschild@sportklinik.de (M.H.); 2 Department of Orthopaedics, University Medicine Greifswald, Ferdinand-Sauerbruch-Straße, 17475 Greifswald, Germany

**Keywords:** sports activity, young patients, THA, MCID, PASS, SCB

## Abstract

Background: The management of degenerative hip diseases in young patients remains a challenge. Despite the improvement of hip-preserving procedures, total hip arthroplasty (THA) may be required in some instances. In addition, young patients undergoing THA have high expectations concerning their postoperative level of activity. Purpose: (1) to define the sports activity level and the return to sports after THA, (2) to describe the modification or initiation of new sports disciplines, and (3) to report the clinically meaningful outcomes after THA in patients younger than 40 years. Methods: A total of 36 patients (40 hips) were prospectively analyzed at a midterm follow-up of 3.9 years. The modified Harris Hip Score (mHHS); the Visual Analog Scale (VAS) for pain; the University of California, Los Angeles (UCLA) activity scale; and sports and recreational activity levels were assessed via questionnaire. The minimal clinically important difference (MCID) was determined by calculating half of the standard deviation, and the substantial clinical benefit (SCB) as well as patient acceptable symptomatic state (PASS), were calculated by the anchor method for the mHHS. Results: At the final follow-up, there was a significant improvement in mHHS (34.1 to 92.6; *p* < 0.0001), UCLA (3.2 to 7.6; *p* < 0.0001), and VAS for pain (8 to 1; *p* < 0.0001). More patients were active in sports at follow-up than before surgery (44% to 92%, *p* < 0.0001). In addition, the duration and frequency of sports activities showed a significant increase (*p* < 0.0001). The MCID, SCB and PASS for mHHS were 89% and 58%, respectively. No revision surgery had to be performed. Conclusion: This study showed that a large proportion of patients under 40 years of age who underwent THA increased their physical activity. Eighty-six percent of the patients were highly active, with a UCLA score ≥ 7. Furthermore, the reported MCID, SCB, and PASS for mHHS were achieved by more than 80% of patients.

## 1. Background

The management of degenerative hip diseases in young patients remains a challenge. Current conditions, such as femoroacetabular impingement syndrome (FAIS), developmental dysplasia of the hip (DDH), and trauma are extensively recognized as sources of pain and functional limitations in active individuals that precede the development of hip osteoarthritis [1,2,3,4,5,6,7]. In the past several years, there has been a rapid increase in the development of surgical techniques to preserve native hip joints, with the appreciation that most hip problems in young adults are associated with altered hip morphology [1,8]. Despite the improvement of both open and arthroscopic hip-preservation procedures, these procedures may not provide adequate symptom relief in the case of severe osteoarthritic changes, and total hip arthroplasty (THA) may be required [8,9,10]. These cases are often especially challenging due to deformity, muscle wasting, and scarring from previous surgeries [11,12]. THA offers young patients the opportunity to achieve excellent pain relief levels and enhanced function. However, young patients undergoing THA often have high expectations concerning their postoperative level of activity [10,13,14]. A systematic review on the functional outcomes after THA among patients under 30 years has been conducted [10]. However, there is a lack of data answering questions about functional outcomes and sports activity levels after THA in young patients.

Therefore, this study’s purpose was (1) to analyze the return-to-activity rate and the physical activity of patients younger than 40 years undergoing cementless THA, (2) to describe the modification or initiation of new sports disciplines, and (3) to report the clinically meaningful outcomes after THA in patients younger than 40 years. We hypothesized that most patients under 40 years treated by THA would be able to return to regular sports and recreational activity.

## 2. Methods

### 2.1. Patient Selection

The present study included a consecutive series of patients younger than 40 years following primary cementless THA performed in a multisurgeon series between January 2008 and December 2018 at our institution. The inclusion criteria were the presence of osteoarthritis, failure of conservative therapy, primary cementless THA implantation, and age at surgery between 18 and 40 years. The exclusion criteria were primary hybrid or cemented THA, revision surgery, age at surgery <18 and >40 years, and refusal to participate in the study. A total of 5.632 patients obtained a THA in this period of time. According to the exclusion criteria, 5.590 patients were excluded. Of the remaining 42 patients, six refused to participate, leaving 36 patients (40 hips) to be included in the final analysis at a mean follow-up of 3.9 ± 1.3 (2–9.5) years. The 6 patients who refused to participate were interviewed by telephone regarding THA survival. None of the 6 patients required a revision.

A cementless hydroxyapatite (HA)-coated titanium femoral stem (Corail stem, DePuy Orthopedics, Warsaw, IN, USA) was used in 36 hips. The other four patients received a cementless neck-preserving hip stem (Nanos, Smith and Nephew, Watford, UK), a cone prosthesis stem (Wagner cone prosthesis stem, Zimmer, Warsaw, IN, USA), a dual taper stem (SL-Plus MIA, Smith and Nephew, Watford, UK), and a short hip stem (Fitmore, Zimmer, Warsaw, IN, USA).

A cementless Pinnacle acetabular cup (DePuy Orthopedics, Warsaw, IN, USA) was used in 30 hips, and a cementless Allofit acetabular cup (Zimmer, Warsaw, IN, USA) was used in 10 displastic hips.

**Figure 1:**
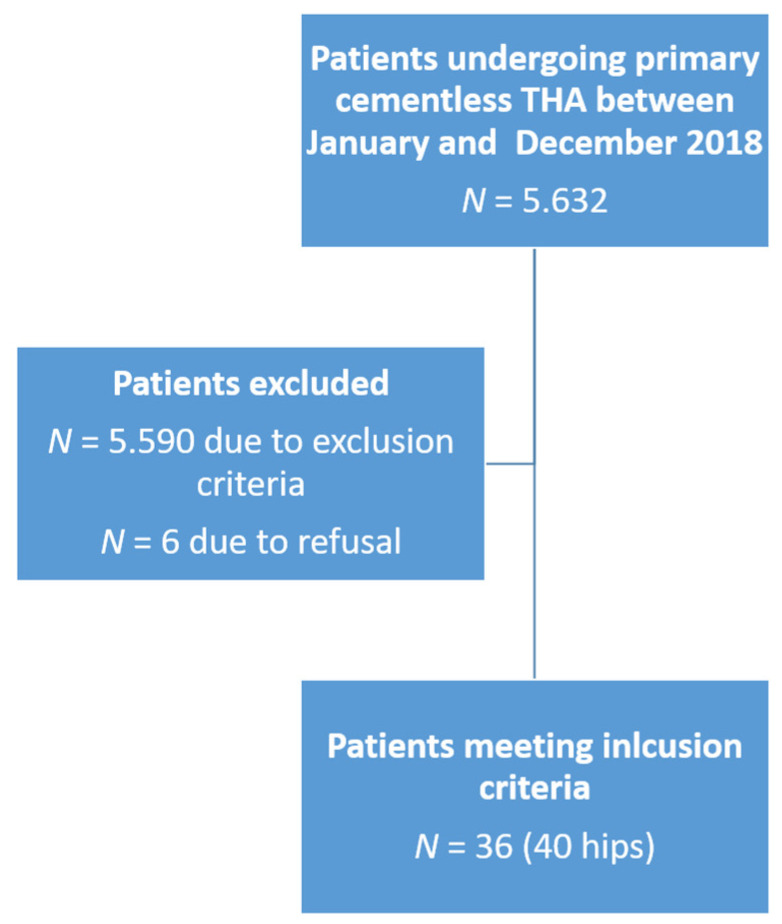
Patient inclusion/exclusion flowchart.

All patients gave informed consent. The local ethics committee approved all procedures (Ethikkommission der Landesärztekammer Baden-Württemberg, Germany, F-2019-006), and the study was conducted following the principles described in the 1975 Declaration of Helsinki, as revised in 2008. The patient enrollment flowchart is shown in Figure 1.

### 2.2. Psychometric Analyses

The modified Harris Hip Score (mHHS) [15] was assessed preoperatively and at the latest follow-up. The patient’s physical activity was assessed using the University of California Los Angeles Activity Scale (UCLA) [16]. The pain level was appraised using the Visual Analog Scale (VAS) for pain. Sporting and physical activities were assessed using the Schultheses Clinic Activity Questionnaire [17]. The sports ability was recorded before the occurrence of the first symptoms and at the follow-up time.

To quantify the clinical significance of meaningful outcome achievement, the minimal clinically important difference (MCID; defined as the smallest outcome difference that the patient perceives as clinically important [18,19]), the substantial clinical benefit (SCB; defined as the clinical value that the patient considers to be considerable and is also seen as the upper limit to the MCID [10,20]) net change, and the patient acceptable symptomatic state (PASS; defined as the postoperative threshold above which a patient is suspected to have had a satisfactory outcome [18]) were calculated for mHHS.

The MCID for mHHS was calculated using half a standard deviation (distribution-based) method.

The SCB net change was calculated at the latest follow-up, where the patients were asked the following anchor question [20,21]: “Since your total hip arthroplasty, how would you rate your overall physical ability?” Patients’ answer choices were “much worse”, “worse”, “slightly worse”, “no change”, “slightly improved”, “improved”, and “much improved”. Patients who responded with “slightly worse”, “no change”, or “slightly improved” were used as the control group. The corresponding difference between “no change” and “much improved” was used to define the SCB.

PASS was also calculated at the latest follow-up by use of the following anchor question [22,23]: ‘‘Taking into account all the activities you engage in during your daily life, your level of pain, and your functional impairment, do you consider that your current state is satisfactory?’’ The SCB and PASS for mHHS were calculated using receiver operating characteristic (ROC) curve analysis [24], and an area under the curve > 0.8 was considered predictive of patients who did and did not achieve SCB and PASS. The cutoff point was defined using Youden’s Index [24].

Descriptive statistics for all continuous variables are reported as the means ± standard deviations. Categorical variables are reported using count and percentage. Differences between preoperative and postoperative data were examined with a t-test and Wilcoxon signed-rank test. McNemar’s test statistic was conducted to detect differences. Statistical analyses were conducted using SPSS statistical software (IBM SPSS Statistics for Windows, version 26.0.0; IBM Corp, Armonk, NY, USA).

## 3. Results

### 3.1. Demographics

Thirty-six patients (40 hips) were included in the analysis. The mean age was 31.5 ± 5 (19–39) years, the mean body index (BMI) was 27.1 ± 5.3 (17.3–43.8) kg/m^2^, the mean duration of surgery was 73.5 ± 25.5 (45–141) minutes, the mean duration of the symptoms prior to surgery was 4.8 ± 4.4 (0.5–23) years, and the mean follow-up was 3.9 ± 1.3 (2–9.5) years. Surgery was performed on 23 men and 13 women. The diagnoses leading to arthroplasty were secondary osteoarthrosis due to FAIS in 6 hips, DDH in 19 hips, trauma in 10 hips, and avascular necrosis of the femoral head (AVN) in 5 hips. Twenty THAs were performed on the right side, and twenty were performed on the left side (Table 1).

At the latest follow-up, there was no revision surgery. However, two patients presented a leg length difference of 1 cm.

### 3.2. Sports and Recreational Activity

**Figure 2:**
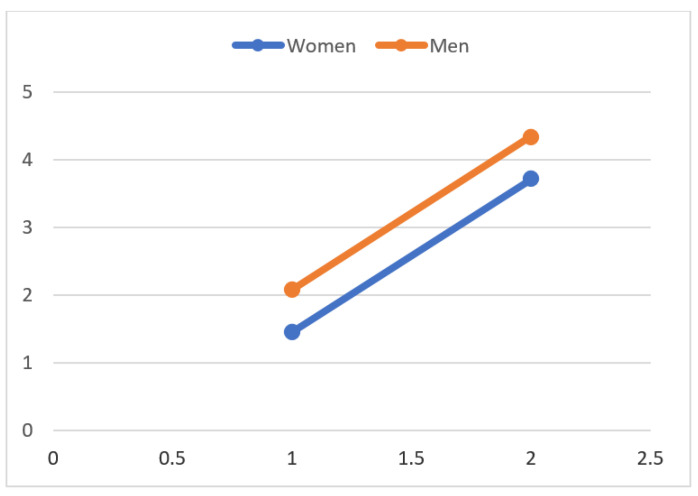
The number of sport disciplines practiced before onset of the first symptoms and after cementless THA. Women increased from 1.4 to 3.7 (*p* < 0.0001), and men increased from 2.1 to 4.4 practiced disciplines (*p* < 0.0001). The values are shown as mean values.

Complete questionnaire data regarding recreational activity were available for 36 patients. After surgery, 33 of 36 patients (92%) were active in at least one recreational activity, compared to 16 of 36 (44%) before the onset of first symptoms. Most of the patients who were inactive started new sports and recreational activities postoperatively. Patients engaged in an average of four different sport disciplines at the last follow-up, which differed significantly from the number of sport disciplines practiced before the onset of the first symptoms (1.8 disciplines; *p* < 0.0001). This increase was confirmed in the individual analyses of men and women. In the comparison between men and women within the groups, there was no difference in the practiced sports disciplines before onset of the first symptoms and after THA (prior: 2.1 to 1.4; *p* = 0.103; after: 4.3 to 3.7; *p* = 0.525) (Figure 2).

Sixty-seven percent of the patients initiated sports and recreational activities within three months after surgery, 17% between 3 and 6 months after surgery, and 17% six months after surgery. Overall, there was a significant increase in the types of sports performed (Table 2 and Table 3). These activities are consistent with the Hip Society member’s consensus guidelines and the American Association of Hip and Knee Surgeons, who have determined which sports are listed as low-, middle-, or high-impact activities, and do not manifest patient goals and expectations [25].

### 3.3. Return to Sports

**Figure 3:**
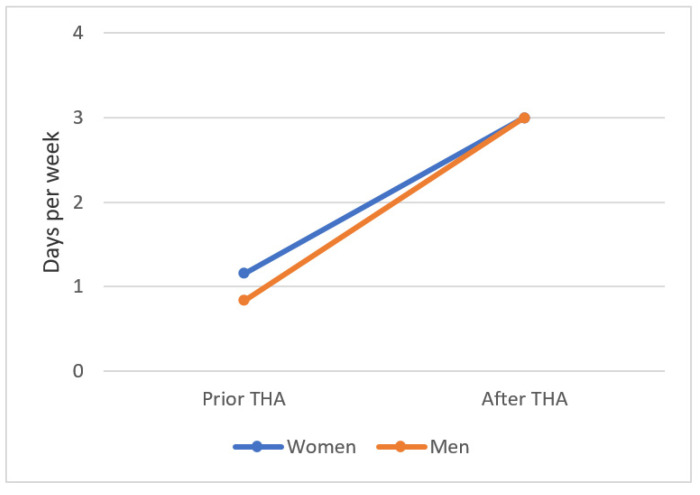
The number of sports sessions per week before onset of the first symptoms and after cementless THA. Women increased from 1 to 3 (*p* = 0.002), and men increased from 0.8 to 3 days per week (*p* < 0.0001). The values are shown as the mean values.

The frequency (sports sessions per week) increased significantly from the level before the onset of the first symptoms to the last follow-up: patients were active one day per week before the onset of the first symptoms and three times per week after THA (*p* < 0.0001). In the analysis of the subgroups, male and female patients participated in sports at the same frequency (three times per week) as the last follow-up (*p* = 0.869) (Figure 3).

**Figure 4:**
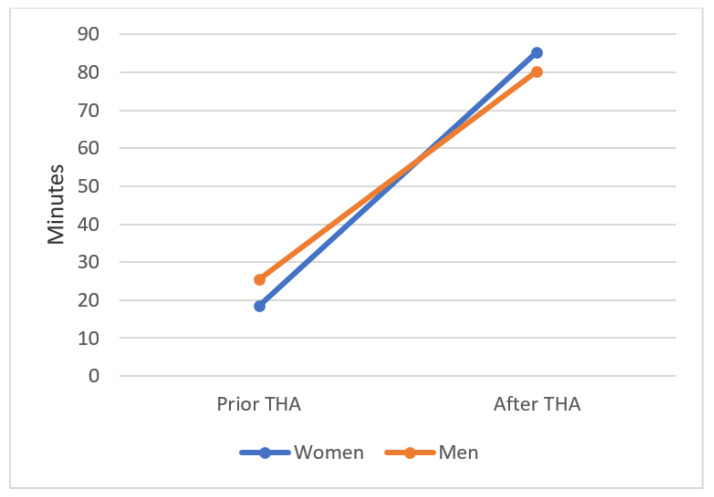
Session length per week of each sport before onset of the first symptoms and after cementless THA. Women increased from 18.4 to 85.4 min, and men increased from 25.4 to 80.2 min. The session length increased significantly. Values are shown as the mean values. *p* < 0.0001.

The minimum session length per week increased from 23 ± 31.6 (0–120) minutes before onset of the first symptoms to 82 ± 40.8 (0–150) min at the last follow-up (*p* < 0.0001). Before onset of the first symptoms, women exercised 18.4 ± 33.1 min, and men exercised 25.4 ± 31.2 min per session. Postoperatively, there was a significant increase in the number of minutes per session practiced; women exercised 85.4 ± 34.3 min, and men exercised 80.2 ± 47.3 min (*p* < 0.0001). There was no postoperative significant difference in session length per week between male and female patients (*p* = 0.716) (Figure 4).

Eighty-five percent of the patients reported an enhancement in sports and recreational activity due to THA.

### 3.4. Outcome Scores

Paired *t*-test analysis of preoperative and postoperative reported outcomes demonstrated statistically significant improvements in mHHS (34.1 ± 18.6 vs. 92.6 ± 12.3; *p* < 0.0001), UCLA (3.2 ± 1.9 vs. 7.6 ± 1.5; *p* < 0.0001), and VAS (8 ± 1.7 vs. 1 ± 1.7; *p* < 0.0001) scores. Postoperatively, 31 patients (86%) reported a UCLA score ≥7, corresponding to being highly active in sport activities (Table 4).

### 3.5. Achievement of MCID, SCB, and PASS

The MCID threshold scores of mHHS was 20. Postoperatively, a total of 32 patients (89%) achieved MCID for the mHHS. The SCB threshold scores of mHHS was 52.8. Postoperatively, a total of 21 patients (58%) achieved SCB for the mHHS. The PASS threshold scores were 85. Postoperatively, a total of 28 patients (78%) achieved PASS for the mHHS.

## 4. Discussion

The present study demonstrated that patients under 40 years of age who underwent THA were highly satisfied with their sports and recreational activity. Eighty-six percent of the patients were highly active, with a UCLA score ≥7. Ninety-two percent of patients were active in at least one recreational activity after THA. Furthermore, these patients showed an improvement and increase in the sports sessions per week (3 days per week) and the session length per week (82 ± 40.8 min). The reported MCID, SCB, and PASS for mHHS were achieved by more than 80% of patients.

Historically, THA has been associated with improvement of patient-reported outcome measures (PROMs), and the development of surgical techniques and implants has led to an increase in patient expectations and postoperative improvement, with substantial attention given to returning to or starting sports [26]. However, data on the activity level and PROMs after cementless THA in patients younger than 40 years are sparse in the literature.

In our cohort, a high rate of return to and an initiation of sports activities was found at the last follow-up. Forty-four percent of the patients actively participated in sports before the onset of the first symptoms, and 92% were active or commenced sports at midterm follow-up. Consequently, 47% of the patients initiated new sports activities. In addition, 85% of patients reported that THA improved sports activity. These data are analogous to the numbers published on uncemented THA [10] and resurfacing hip arthroplasty in patients with a mean age of 52.6 years [27]. In our cohort, before onset of the first symptoms, only a few patients were practicing high-impact sports, such as jogging, volleyball, and skiing. Interest and participation in low-impact sports, such as hiking, fitness training, and biking significantly increased (up to 250%) after surgery, in accordance with previous studies [28,29,30]. However, postoperatively, an increase in both high- (up to 200%) and low-impact sports was observed, while no reports of high-impact sports are available in the literature [13,29,31,32]. According to AHKS guidelines, the practice of low-impact sports is recommended without any previous sports experience or supervision, while medium- or high-impact sports are recommended only with previous sports experience or supervision. In accordance with the AHKS guidelines, most of our patients practiced low-, medium-, or high-impact sports. However, 33% of our patients practiced sports that were classified as not recommended even with or without previous experience or supervision (jogging, contact sports, and high-impact aerobics). Based on our own experience, we do not recommend or prohibit specific sports disciplines. Nonetheless, patients are informed of general and sport-specific risks of higher activity and impact levels, such as possible increased wear or earlier implant failure, among others. However, we observed a conversion of low-impact to intermediate/high-impact sports practices after surgery.

Regarding PROs, in the general population, the mean postoperative mHHS at one-year follow-up was 88.6 (preoperative 50.8) points [33]. In our cohort population, this score improved significantly to 92.6 (preoperative 34.1) points at the last follow-up. A systematic review of THA in young patients (mean age 30.9 years) showed a mean postoperative Harris Hip Score (HHS) of 84.6 at the 7.4-year follow-up [34]. Overall, a significant increase in PROMs was demonstrated [31,33,35]. We were thus able to confirm these very good clinical results for the young patient cohort. However, considering the level of activity based on the UCLA score, a large proportion of our patients (86%) were highly active after THA (UCLA score ≥ 7). These results are in agreement with the reported values in the literature [31].

### 4.1. Limitations

Our study is not free of limitations. First, the small cohort size may be explained by the new development of joint-preserving surgery. Second, there is heterogeneity in terms of implants used and indications for THA. Due to the small number of cases, however, no further subgroups could be formed. Third, there are currently no preliminary studies in the literature reporting these clinically meaningful outcome scores, as well as sport and recreational activity levels in very young patients. The outcomes from this study were derived from self-reported data. However, scores, such as the mHHS, demonstrated a ceiling effect [10,36,37]. The implication is that patients may report very high scores and still suffer some functional deficits. This suggests that additional tools to characterize young active patients undergoing THA are needed in the future. Last, it should be noted that potential subgroups may have different preoperative sport levels. For example, DDH patients may not have performed high-impact sports due to hip problems, so a higher level of gain was reported after THA. However, due to the small group size, this effect could not be analyzed further.

### 4.2. Conclusions

A large number of patients 40 years or younger who underwent THA returned to sports, and their sporting ability increased significantly. Eighty-six percent of the patients were highly active with a UCLA score ≥7. Furthermore, the reported MCID, SCB, and PASS for mHHS were achieved by more than 80% of our patients.

## Figures and Tables

**Table 1:** Patient demographic data.

	Value
Total no. of patients	36
Laterality, *n* (%)	
Right	20 (50%)
Left	20 (50%)
Sex, *n* (%)	
Female	13 (36%)
Male	23 (64%)
Age, years	31.5 ± 5 (19–39)
Body mass index, kg/m^2^	27.1 ± 5.3 (17.3–43.8)
Follow-up, years	3.9 ± 1.3 (2–9.5)
Preoperative-diagnoses, *n* (%)	
FAIS	6 (15%)
DDH	19 (47.5%)
Trauma	10 (25%)
Avascular necrosis of the femoral head	5 (12.5%)

Values are shown as *n* (%) or mean ± SD (range). DDH, developmental dysplasia of the hip; FAIS, femoroacetabular impingement syndrome.

**Table 2:** Sports disciplines before onset of the first symptoms and after THA.

Discipline	Prior to THA, *n* (%)	After THA, *n* (%)	*p*-Value
Long Walks	8 (22.2%)	27 (75%)	<0.0001
Biking	12 (33.3%)	29 (80.6%)	<0.0001
Hiking	5 (13.9%)	21 (58.3%)	<0.0001
Nordic-Walking	1 (2.8%)	6 (16.7%)	0.063
Fitness Training	6 (16.7%)	23 (63.9%)	<0.0001
Alpine skiing	4 (11.1%)	6 (16.7%)	0.500
Jogging	2 (5.6%)	8 (22.2%)	0.031
Soccer	2 (5.6%)	2 (5.6%)	0.500
Handball	2 (5.6%)	2 (5.6%)	0.500
Volleyball	1 (2.8%)	3 (8.3%)	0.250
Golf	1 (2.8%)	1 (2.8%)	0.500

Values are shown as *n* (%).

**Table 3:** Sports disciplines before onset of the first symptoms and after THA.

Discipline	Women			Men		
	Prior to THA, *n* (%)	After THA, *n* (%)	*p*-Value	Prior to THA, *n* (%)	After THA, *n* (%)	*p*-Value
Long Walks	3 (8.3%)	12 (33.3%)	0.004	5 (13.9%)	15 (41.7%)	0.002
Biking	4 (11.1%)	11 (30.6%)	0.016	8 (22.2%)	18 (50%)	0.002
Hiking	1 (2.8%)	5 (13.9%)	0.125	4 (11.1%)	16 (44.4%)	<0.0001
Nordic-Walking	0	3 (8.3%)	0.250	1 (2.8%)	3 (8.3%)	0.500
Fitness Training	0	10 (27.8%)	0.002	6 (16.7%)	13 (36.11%)	0.016
Alpine skiing	0	0	1.000	4 (11.1%)	6 (16.67%)	0.500
Jogging	1 (2.8%)	2 (5.6%)	1.000	1 (2.8%)	6 (16.67%)	0.063
Soccer	0	0	1.000	0	2 (5.6%)	0.500
Handball	1 (2.8%)	1 (2.8%)	1.000	1 (2.8%)	1 (2.8%)	0.500
Volleyball	0	1 (2.8%)	1.000	1 (2.8%)	2 (5.6%)	0.500
Golf	0	0	1.000	1 (2.8%)	1 (2.8%)	0.500

Values are shown as *n* (%); THA, total hip arthroplasty.

**Table 4:** Pre- and postoperative patient-reported outcomes.

Score	Preoperative	Postoperative	*p*-Value
mHHS	34.1 ± 18.6 (3.3–81.4)	92.6 ± 12.3 (41.8–100)	<0.0001
UCLA	3.2 ± 1.9 (1–8)	7.6 ± 1.5 (3–10)	<0.0001
VAS	8 ± 1.7 (3–10)	1 ± 1.7 (0–7)	<0.0001

Values are shown as *n* (%) or mean ± SD (range). mHHS, modified Harris Hip Score; UCLA, the University of California and Los Angeles activity scale; VAS, Visual Analog Scale for pain.

## Data Availability

The data presented in this study are available on request from the corresponding author.
